# Management of Scaphoid Nonunion (SNU) With Ilizarov Ring Fixator Using Two Olive Wire Compression Without Bone Grafting: A Case Series

**DOI:** 10.7759/cureus.31646

**Published:** 2022-11-18

**Authors:** Mofakhkharul Bari, Srikant Konchada, Sandeep Pradhan, Ankit Gulia, Bodanapu Sandeep, Divyadeep Goyal, sumit kaushik, Kashish Dhingra

**Affiliations:** 1 Orthopaedics, Bari-Ilizarov Orthopaedic Centre, Dhaka, BGD; 2 Orthopaedics, Kalinga Institute of Medical Sciences, Bhubaneswar, IND; 3 Orthopaedics, Kalinga Institute of Medical Sciences, Bhubaneshwar, IND; 4 Orthopaedics and Traumatology, Kalinga Institute of Medical Sciences, Bhubaneswar, IND

**Keywords:** ilizarov fixation for non union and infection, management scaphoid fracture, outcome analysis, non-union, ilizarov fixator, scaphoid nonunion

## Abstract

Background: The scaphoid is the most commonly fractured bone among the carpal bones seen in orthopedic practice. The majority have good favorable prognosis, but some develop nonunion of fracture despite optimal treatment, which can lead to further complications if left untreated. It is recommended that displaced scaphoid nonunions (SNUs) should be reduced and fixed to prevent degenerative changes from occurring, even if they are asymptomatic. Many treatments have been described, from a percutaneous fixation with a k-wire or screw to open reduction and internal fixation with or without bone grafting, but none of them is the gold standard.

Aim: To evaluate the outcome of an SNU treated with an Ilizarov fixator using two olive wires without bone graft.

Methods: Eleven cases of non-union scaphoid fractures were considered in the study which was presented to the Department of Orthopedics of Kalinga Institute of Medical Sciences during the period of March 2015 to March 2018. This study has been approved by the scientific and ethical committees. The anatomical location of the fracture was graded according to the MAYO classification. An Ilizarov frame was applied with two cross olive wires for achieving compression at the fracture area and maintained for six weeks. A final outcome was assessed using the scaphoid outcome score.

Results: One out of 11 patients operated on during the study period was lost in follow-up, so 10 patients were considered for analysis of the results. There were nine male patients and one female patient. The majority were right-sided and dominant-handed, with varied occupations. The average duration of nonunion, when presented, was 10.7 months (a range of 6-20 months). The average follow-up was 43.6 months (range 27-60 months). Union was achieved in an average of 12.9 weeks (range 10-18 weeks). All the patients returned to their pre-injury activity level in a mean of 17.1 weeks (range 13-23 weeks). Grip strength improved from a mean of 29.5 kg preoperatively to 39.4 kg postoperatively. At the final follow-up, the mean scaphoid outcome score was 9.1. An excellent outcome was obtained in five cases (50%), a good outcome in three cases (30%), a fair outcome in one case (10%), and in one case (10%), a poor outcome.

Conclusion: With our technique of Ilizarov fixation and compression with two cross olive wires, SNU can be treated safely even without opening the non-union site and even without bone grafting. Since we excluded SNU patients with humpback deformity, carpal instability, carpal collapse, or avascular necrosis (AVN), our results might not be directly comparable to those of other SNU series in the literature. These would have predisposed to a poor outcome. Since we did not assign the patients at random, it is challenging to compare the Ilizarov technique to other widely used SNU treatments and determine whether it is more effective. However, the study's results are encouraging and show that the Ilizarov method using two olive wires for compression.

## Introduction

Scaphoid fractures mostly result from a fall on an outstretched hand with forced dorsiflexion, and it is the most commonly fractured carpal bone [1-2]. Precarious blood supply is the main one of many factors contributing to the high incidence of nonunion despite treatment. However, with appropriate treatment, the nonunion rate becomes low [3]. Other risk factors for nonunion include the degree of fracture displacement, delayed or missed diagnosis, inadequate treatment, and fracture location [4].

Scaphoid nonunion (SNU) can be asymptomatic initially, but the natural history of untreated SNU progresses to degenerative and arthritic changes in adjacent intercarpal joints, leading to impairment of wrist joint function and affecting activities of daily living [5]. Non-displaced SNUs are also recommended to be treated and fixed to prevent degenerative changes from occurring, even if they are asymptomatic [6].

Many treatments have been suggested, from a percutaneous fixation with a k-wire or screw to open reduction and internal fixation with or without bone grafting, but none of them is the gold standard [4, 7].

The Ilizarov technique has been used to close gaps in long bones that do not join together and fix deformities by using mechanical forces to make new bone and improve blood flow [8-9]. Treatment for nonunion with the Ilizarov approach has grown in popularity. Ilizarov procedures primarily use three techniques: compression osteosynthesis, acute compression and lengthening, and bone transport. For minor gaps or no deformities, compression osteosynthesis is appropriate.

This study will look at what happens when a nonunion of a broken scaphoid is treated with an Ilizarov fixator and olive wire compression at the broken site instead of a bone graft.

## Materials and methods

Some 11 cases of SNU fractures were considered in the study that was presented to the Department of Orthopaedics of Kalinga Institute of Medical Sciences during the period of March 2015 to March 2018. This study has been approved by the scientific and ethical committees with IRB approval number KIITS/012/OR2022. After getting their consent to take part in the study, subjects who met the inclusion and exclusion criteria were added to the study (Table 1).

**Table 1:** Inclusion and exclusion criteria. SNU, scaphoid nonunion

Inclusion criteria
Clinically and radiologically established cases of SNU
Mayo classification: scaphoid fractures in the middle third
A minimum of six months must elapse after the initial injury
Immobilization as the primary treatment, neglected cases, late or missed diagnosis
Exclusion criteria
Fresh fractures of the scaphoid
Other fractures, dislocations, ligament injuries, or instability in the same wrist
Degenerative changes/arthritis of the wrist
Cases of previously operated SNU

Eleven subjects were operated on and included in the study. Meeting the above criteria, they were evaluated with plain radiographs with antero-posterior, scaphoid and oblique views to demonstrate SNU with smooth, rounded, sclerosed fracture margins. The anatomical location of the fracture was graded according to the Mayo classification [10]. Patients' complaints like wrist pain, decreased strength, and inability to do normal work were noted. Before surgery, the patient's dominant hand, job, range of motion, and grip strength at the wrist were also taken into account.

A final outcome was assessed using the scaphoid outcome score [11-12], taking pain, function, motion, and overall satisfaction into consideration, and a final score was tabulated as the sum of individual parameters and was further categorized either as excellent, good, moderate, or poor depending on the final scoring system.

Surgical technique with an Ilizarov fixator

Patients were operated on under regional anesthesia without applying tourniquets. The operated wrist is placed on the arm table in a supine position under a C-arm fluoroscopy unit that is placed across the table perpendicular to the wrist. Two ring Ilizarov frames were applied, spanning the wrist joint. The proximal ring was placed 4-6 cm proximal to the wrist joint with the use of two biocompatible Ilizarov wires of 1.5 mm thickness, one passing through the ulna and another through the radius (from the volar to the dorsal aspect at a 30-degree angle) and ulna (30-45 degrees at an angle from the dorsal to the volar aspect, and came out from the skin 2-3 mm from the tendon of the flexor carpi ulnaris muscle) in a safe anatomical zone after tensioning the wire to 90-100 kg load. 

**Figure 1:**
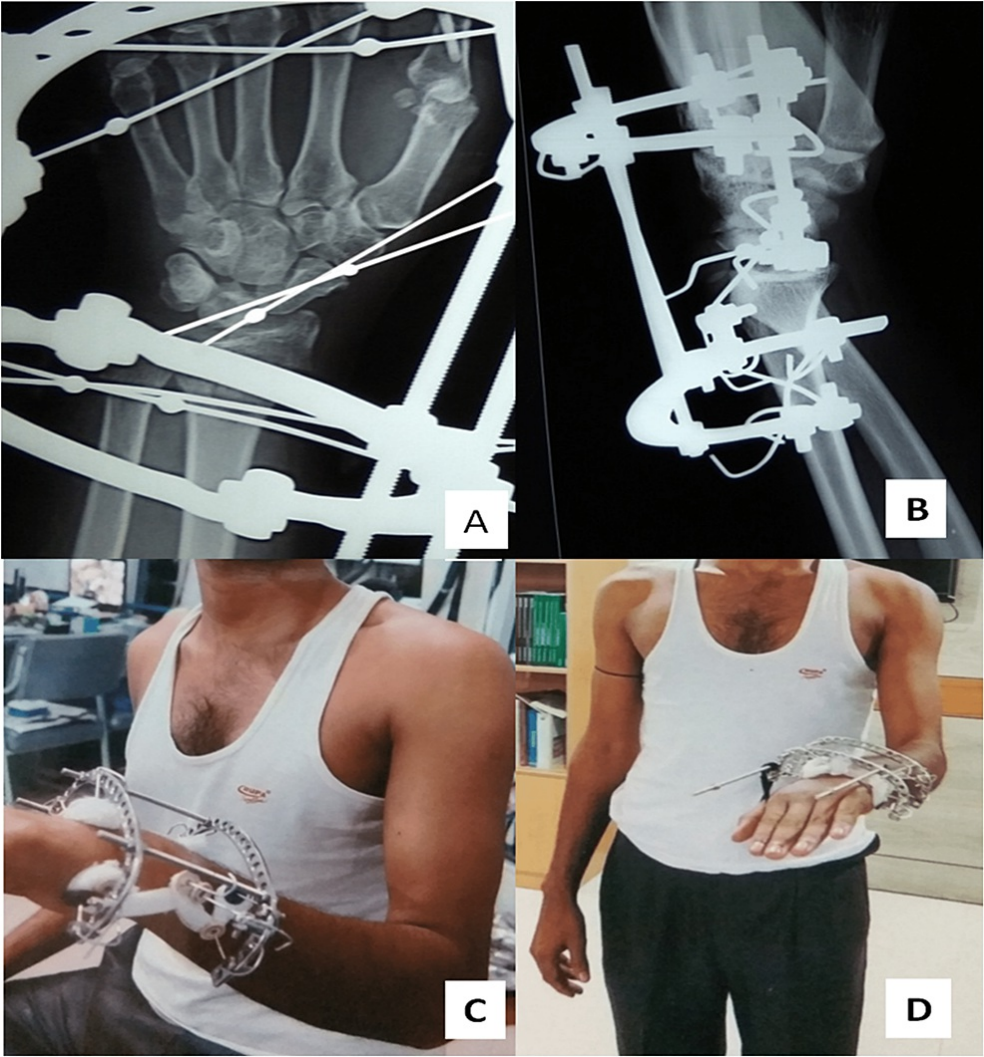
An illustration of the Ilizarov device applied across the wrist using two olive wires through scaphoid (A-D) in patient number 1.

Similarly, the distal ring is applied to the mid metacarpal level by attaching to two similar wires of 1.5 mm, one passing from the radial border of the second metacarpal exiting from the third metacarpal and another passing from the ulnar border of the fifth metacarpal exiting from the fourth metacarpal at an angle of 30-40 degrees. One 1.5-mm olive wire is passed through the nonunion site from the proximal to the distal pole of the scaphoid bone, with the olive stopping at the proximal pole, and the sharp end of the wire is attached to the distal ring with a male post. Another similar olive wire is passed through the scaphoid from its distal pole, exiting the proximal pole obliquely through nonunion, with the olive stopping at the distal pole of the scaphoid bone. The sharp end of this wire is attached to the proximal ring with a wire fixation bolt. Before fixing, these two cross wires are gradually tensioned until the SNU site is compressed under fluoroscopy without opening the nonunion site at all. Four connecting rods are also fixed between the two rings (Figure 1A-D).

Postoperative evaluation

After the immediate post-operative radiograph, weekly serial radiographs were taken until the frame was taken off at a duration of six weeks to look for nonunion compression, wire detensioning, and pin tract osteolysis, and these problems were dealt with as needed. Every week, the fixator's hardware was checked for stability, and loose nuts were tightened on a regular basis. The finger small joints, elbow, and shoulder joints were all encouraged to move. The frame was removed at six weeks. Patients were encouraged to do an active range of motion at the wrist to overcome post-immobilization stiffness. Patients were evaluated clinically and radiologically at two-weekly intervals following surgery until the bony union was demonstrated on radiographs. They were also evaluated clinically at 6, 12, and 24 months after frame removal to look for any collapse, avascular necrosis with the fragmentation of the proximal pole, and arthritic changes. Grip strength at the final follow-up was measured with a Jamar analog hand dynamometer and compared with preoperative values. With the help of the scaphoid outcome score, the functional outcome was measured by comparing pain, function, range of motion, and overall satisfaction before and after surgery.

## Results

One out of eleven patients operated on during the study period was lost in follow-up, so 10 patients were considered for analysis of the results. There were nine male patients and one female patient. The majority were right-sided and dominant-handed, with varied occupations. The average duration of nonunion, when presented, was 10.7 months (range 6-20 months). Eight patients were initially treated with cast immobilization; the remaining two patients were fully neglected and did not receive any form of treatment (Table 2).

**Table 2:** The patient's demographic characteristics. D, dominant hand; ND, non-dominant hand

S. no.	Sex	Side/dominant	Age (years)	Occupation	Mechanism of injury	Nonunion duration (months)	Initial treatment
1	Male	Right/D	25	Student/sports	Sports	6	Cast
2	Male	Right/D	18	Student	Sports	9	Cast
3	Male	Left/ND	20	Student	Fall from bike	7	Cast
4	Female	Left/D	30	Office	Fall from staircase	11	Cast
5	Male	Right/D	28	Manual labor	Fall	20	Neglected
6	Male	Left/ND	35	Plumber	Fall	8	Cast
7	Male	Right/D	22	Student	Fall from bike	6	Cast
8	Male	Right/D	27	Driver	Fall	7	Cast
9	Male	Right/D	30	Electrician	Fall	18	Neglected
10	Male	Right/D	32	Manual labor	Fall	15	Cast

**Figure 2:**
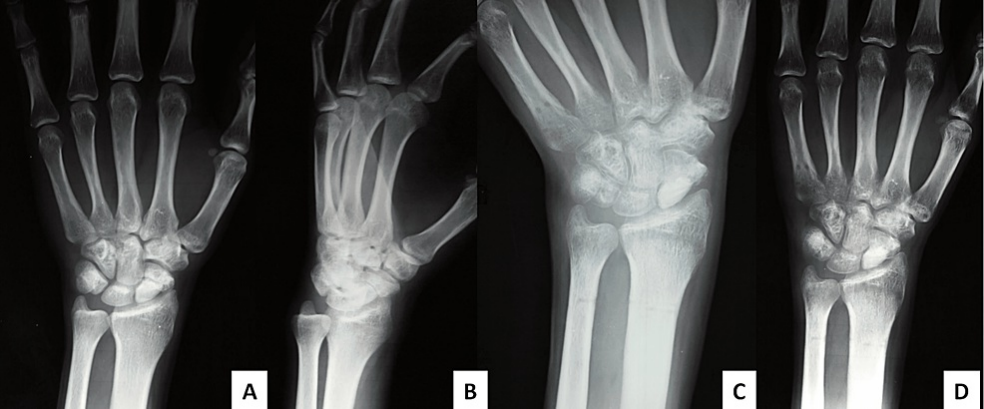
SNU in patient number 1, a preoperative radiograph (A,B) and a postoperative X-ray of the healed SNU in patient number 1 (C,D). SNU, scaphoid nonunion

**Figure 3:**
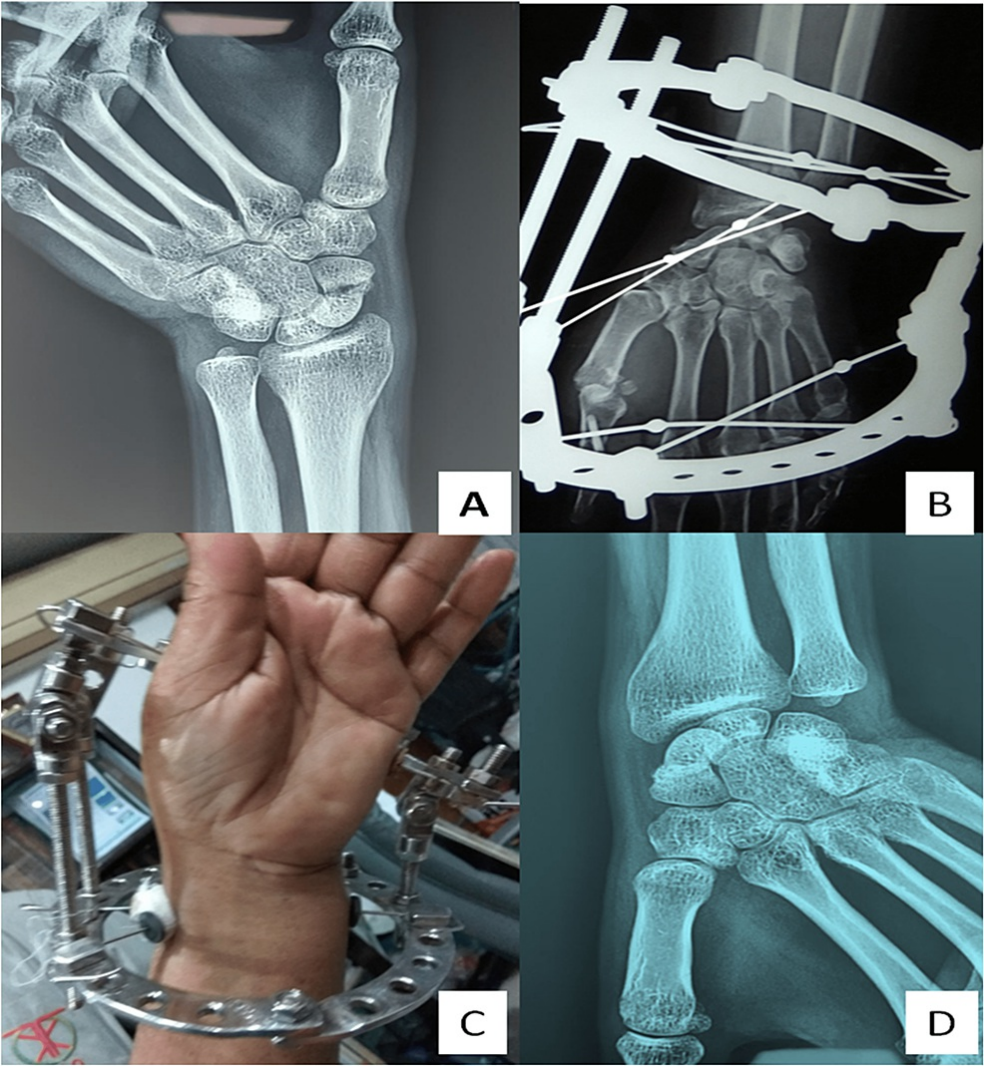
SNU in patient number 4, a preoperative radiograph (A) and a postoperative X-ray (B) and healed SNU radiograph in patient number 4 (D). SNU, scaphoid nonunion

The average follow-up was 43.6 months (range 27-60 months). Union was achieved in an average of 12.9 weeks (range 10-18 weeks). All the patients returned to their pre-injury activity level in a mean of 17.1 weeks (range 13-23 weeks). Grip strength improved from a mean of 29.5 kg preoperatively to 39.4 kg postoperatively. At the final follow-up, the mean scaphoid outcome score was 9.1. An excellent outcome was seen in five cases (50%), a good outcome in three cases (30%), a fair outcome in one case (10%), and in one case (10%), a poor outcome (Table 2). Three cases had experienced pin tract infection (PTI) at metacarpal pins that were treated with regular local normal saline dressings and oral antibiotics, and as they were responding well to the above treatment regimen and were found to be stable, they were retained for six weeks. No problems like broken hardware, nerve damage, or tendons getting caught in the wires were reported (Table 3). Representative pre-operative nonunion scaphoid radiographs and post-operative united scaphoid fracture are shown in Figures 2-3.

**Table 3:** Clinical outcomes. PTI, pin tract infection

S. no.	Follow-up duration (months)	Union achieved in weeks	Return to preinjury activity (week)	Flexion in degree	Extension in degree	Grip strength (before/after surgery) in kg	Complications	Post op score	Final outcome
1	60	12	18	70	60	23/38		9	Good
2	58	11	15	80	70	32/43		11	Excellent
3	53	10	13	80	70	33/43	PTI	11	Excellent
4	50	13	16	60	50	32/36		8	Good
5	47	15	22	40	40	23/30		5	Poor
6	44	12	15	70	60	34/45		10	Excellent
7	36	18	23	50	40	32/36	PTI	7	Fair
8	32	11	14	70	70	27/43		11	Excellent
9	29	14	18	80	70	32/41	PTI	10	Excellent
10	27	13	17	70	60	27/39		9	Good
Mean	43.6	12.9	17.1	67	59	29.5/39.4		9.1	

## Discussion

Scaphoid nonunion is difficult to treat due to its precarious blood supply and even more challenging when it has been neglected for a long time. The success rate decreases almost to 62% after a delay of five years [7]. Long-term SNU can cause chronic wrist pain, less range of motion in the wrist, and degenerative changes [11].

Many procedures have been proposed for the treatment of SNU. Bone grafting with anatomical reduction and alignment with stable internal fixation is the main treatment for these. Internal fixation was either with Herbert screws or k-wire fixation [13]. In a meta-analysis [14], Herbert screw fixation with bone grafting achieved a 94% union rate compared with k-wire fixation and bone grafting. Herbert screw fixation has the advantage of early mobilization over k-wire fixation. Vascularized bone grafting has increased the union rates in SNU cases, although it is technically very demanding. Ribak et al. in their study showed that vascularized bone grafting is mandatory in SNU with nonvascular sclerosed proximal fragments and optional when fragments are vascular, depending on the surgeon’s expertise [11]. The Matti-Russe technique is described for the treatment of SNU by filling the nonunion site with cortical and cancellous nonvascularized bone graft without the use of hardware but is complicated by a long duration of immobilization [15]. Whatever treatment option is chosen for SNU, a successful outcome is dependent on achieving the goals of preserving blood supply, stable fixation, bone grafting to bridge the gap, stimulating union, and achieving union before degenerative changes develop [16].

Our method of treating these SNUs with an Ilizarov ring fixator with olive wire compression without the use of bone graft is new and has never been described. The main benefits of our study are that it has all the benefits of not opening the non-union site, like keeping the blood supply going, lowering the risk of infection, preventing soft tissue damage, which can lead to fibrosis, and avoiding bone grafting and the problems that come with it at the donor site.

The study done by Bumbaširević et al. [17] is the only other study in the literature describing the use of the Ilizarov fixator for treating SNU. Our study was different from theirs with the use of two olive wire compressions, which achieved desirable good compression at the nonunion site. Our procedure is technically simpler and easier with no multiple-staged frame adjustment required, unlike the procedure described by Bumbaširević et al. In our study, 80% of cases had good to excellent results, which was on par with their study, and achieved 78% of good to excellent results.

The major disadvantage of our technique was the bulkiness of this external frame. Most of the patients adjusted to that. Like other methods described for the treatment of SNU, immobilization of the wrist was part of the treatment, and it was for six weeks in our study, much less than most of the other studies [18]. No patient had any residual stiffness after frame removal as intense physiotherapy was part of the treatment. Tendon, neurovascular entrapment are possible complications of pins applied for the frame, but we did not encounter any such complications. In Ilizarov fixation, PTIs are common, but we cut down on them by using rubber stoppers [19] and found that in three cases, they went away after a local dressing and oral antibiotics.

Despite the promising results of this new technique, our study has limitations as it is not randomized and we did not compare it with the other established methods of treating such cases. Further studies and randomized controlled trials are needed to establish this as the preferred treatment method as it is difficult to treat SNUs without opening and requires bone grafting.

## Conclusions

With our technique Ilizarov fixation and compression with two cross olive wires, SNU can be treated safely and successfully without opening the nonunion site and even without bone grafting. Our results might not be directly comparable to those of other SNU series in the literature as we did not include SNU cases with humpback deformity, carpal instability, carpal collapse, or avascular necrosis (AVN). These would have predisposed to a poor outcome. It is challenging to determine whether the Ilizarov approach is better because we did not randomly assign the patients and compare it to other well-established treatments for SNU. However, the study's results are encouraging and show that the Ilizarov method using two olive wires compression without bone grafting is an effective procedure. They also suggest that, in some circumstances, it may be a successful means of treating SNU. Further studies are required to establish this as the preferred treatment method in specific situations without associated complications.
